# Gut Inflammation and Immunity: What Is the Role of the Human Gut Virome?

**DOI:** 10.1155/2015/326032

**Published:** 2015-04-07

**Authors:** Alfredo Focà, Maria Carla Liberto, Angela Quirino, Nadia Marascio, Emilia Zicca, Grazia Pavia

**Affiliations:** Department of Health Sciences, Institute of Microbiology, School of Medicine, University “Magna Graecia”, Viale Europa, Germaneto, 88100 Catanzaro, Italy

## Abstract

The human virome comprises viruses that infect host cells, virus-derived elements in our chromosomes, and viruses that infect other organisms, including bacteriophages and plant viruses. The development of high-throughput sequencing techniques has shown that the human gut microbiome is a complex community in which the virome plays a crucial role into regulation of intestinal immunity and homeostasis. Nevertheless, the size of the human virome is still poorly understood. Indeed the enteric virome is in a continuous and dynamic equilibrium with other components of the gut microbiome and the gut immune system, an interaction that may influence the health and disease of the host. We review recent evidence on the viruses found in the gastrointestinal tract, discussing their interactions with the resident bacterial microbiota and the host immune system, in order to explore the potential impact of the virome on human health.

## 1. Introduction

The human virome is essentially a collection of all the viruses that are found in or on human beings. Continuously being updated, the human virome comprises eukaryotic and prokaryotic viruses, viruses that cause acute, persistent, or latent infection, and viruses that can integrate themselves into the human genome, for example, endogenous retroviruses [1, 2].

Both eukaryotic and prokaryotic viruses share lytic or latent life-cycles, which allow different virome/host interactions and promote virus survival and evolution [2]. As a result, human eukaryotic viruses can affect host physiology, mainly when chronically infecting particular sites, and virus-derived genetic elements can modify host gene and protein expression once integrated into host chromosomes [3–5]. Moreover, it has recently been shown that interactions between archaeal viruses and host cells in mammals are comparable with the well-documented relationships that exist between prokaryotic viruses and bacteria [6].

Nevertheless, the size of the human virome is not fully known. As discussed by Mokili et al. [7], our own cells are outnumbered about 10-fold by our bacteriome, and it has been postulated that the number of viruses in our body could be 10-fold higher still. Furthermore, the emerging evidence of new RNA viruses, unknown before the advent of innovative sequencing platforms, suggest that the eukaryotic virome may be far larger than previously thought [8].

The human gastrointestinal tract in particular plays host to one of the most complex microbial ecosystems and an intricate group of viruses. Progress in sequencing technology research is enabling us not only to detect the presence of such microorganisms, but also to evaluate how the intestinal microbiome affects human health. Such approaches have already shown how the gut microbiome, by interacting with the mucus layer, epithelial cells, and underlying lamina propria immune cells, can contribute to the health or disease of the host [9]. It is likely that similar studies into the complex interactions between the resident gut virome and immune and inflammatory processes could shed light on the pathogenesis of intestinal and extraintestinal diseases.

## 2. Human Gut Virome

Human faeces are known to contain at least 10^9^ virus-like particles per gram [10]. Sequencing of gut viruses from faecal samples has shown that bacteriophages, which can harbour up to 10^14^ bacterial cells, are the most prevalent enteric viruses [11]. That being said, as discussed by Minot et al. [12], prokaryotic viruses are almost 10-fold more abundant in the gut than prokaryotes. This indicates that there is a dynamic community structure within the gastrointestinal tract, characterized by predator-prey interactions and thereby providing a source of horizontal gene transfer [13].

Although many gut bacteriophages have not yet been fully classified, the most abundant prokaryotic viruses in the intestine are currently thought to be the tailed, double-stranded DNA viruses of the order Caudovirales (Podoviridae, Siphoviridae, and Myoviridae), together with the tailless, cubic, or filamentous single-stranded DNA viruses (Microviridae) [14]. Prokaryotic viruses are known to influence human health by affecting bacterial community structure and function [12, 15, 16], but the intricate pathways by which this influence is exerted are yet to be fully clarified. Thus far, however, it has been discovered that (i) temperate phages are common; (ii) bacteriophages vary widely between individual hosts but not within a single subject; and (iii) the variety of bacteriophages present increases in adulthood, and the diet affects the composition of phage communities [12].

There are far fewer eukaryotic viruses than bacteriophages in the gut [15, 17, 18]. Nevertheless, sequencing of faecal samples from healthy children has revealed a complex community that includes viruses of the family* Picobirnaviridae*,* Adenoviridae*,* Anelloviridae*,* Astroviridae* and species such as* bocaviruses*,* enteroviruses*,* rotaviruses*, and* sapoviruses *[19]. Despite being fewer in number, these viruses also have significant effects on human health, both in healthy and immunocompromised subjects, causing acute gastroenteritis, acute enteritis, or colitis [19–22]. Picobirnaviruses, for instance, have been found in stool samples from individuals with diarrhoea of unknown aetiology [23–25], as well as in healthy subjects [19], leaving their pathogenic capability up for discussion. Among the RNA viruses found in the gut, a prevalence of plant viruses has been demonstrated, presumably introduced in the diet [12, 17].

Interesting studies have recently described the temporal dynamics of the human gut virome. It appears that the symbiotic relationships between host and virome develop at a young age, with specific variations occurring during the first two years of life, coinciding with environmental and dietary changes. As a result, individuals on the same diet showed similar gut virome composition [12, 17].

Given these findings, it is timely to evaluate the potential role of the gut virome in homeostasis, intestinal immunity, and inflammation.

## 3. Interaction and Recognition between the Virome and the Host Immune System

The enteric immune system exists in a continuous but dynamic equilibrium with all components of the gut microbiome, including the virome [26, 27]. It is likely that this interaction may influence the host's health and disease [2] by modulating the immune system itself [2, 28].

### 3.1. Viromal Effects on the Immune System

The virome is an important regulator of intestinal homeostasis and inflammation [29]. In this regard, as discussed by Foxman and Iwasaki [3], the virome is able to stimulate continuous low-level immune responses without causing any overt symptoms. This capacity has been documented for several systemic viruses, including Herpesviruses and Polyomaviruses, as well as Hepatitis B (HBV) and C (HCV) viruses in some individuals. Given what we know about virus-host interaction at the molecular level, it is feasible that variations within systemic and local gut virome, acting as commensal viruses, could even shape the immunophenotype of the host [2].

### 3.2. Interaction and Recognition between Phages and the Immune System

In addition to the role of the virome in regulating the bacterial microbiome (see below), there is evidence that bacteriophages may also directly interact with the human immune system. For example, as both Duerr et al. [30] and Hamzeh-Mivehroud et al. [31] showed, orally administered phages translocate* in vivo* to systemic tissue, wherein they trigger innate and adaptive immune responses. The humoral immune response induced by bacteriophages has also been documented in several different studies [32–35].

Nonetheless, little is known of the mechanism by which bacteriophages elicit innate antiviral immune responses. In asymptomatic individuals, the dynamic balance between the virome and the intestinal immune system is finely regulated by cytokines secreted by immune cells. These cells are able to recognize antigenic components or pathogen-associated molecular patterns (PAMPs), including those produced by viruses [2]. Toll-like receptors (TLRs) have also been postulated as innate antiviral immune sensors, with TLR3, TLR7, TLR8, and TLR9, as well as RIG-I—a cytoplasmatic double-stranded RNA helicase—and the cytoplasmatic DNA sensor cyclic-GMP-AMP (cGAMP) synthase reportedly involved in the recognition of viral structure. Activation of such receptors triggers signalling cascades that activate the transcription of nuclear factors such as NF-kB, IRF3, and IRF7, which in turn promote the expression of antiviral effectors such as type I interferon, proinflammatory cytokines such as Interleukin-6 and Interleukin-1 beta (*β*), and chemokines such as Interleukin-8 and CXCL-10 [36]. In an asymptomatic host, commensal bacteriophages activate one or more of these pathways, thereby inducing tonic stimulation of the antiviral immune response, and therefore a continuous cycle of cytokine production. These cytokines also exert their action on nonimmune cells and may continuously induce inflammatory processes therein, thereby conferring constant protection against pathogenic viral infections [28, 37].

Another mechanism by which bacteriophages interact with the immune system is through their association with the bacterial microbiome. Some bacteriophages use commensal bacteria as a vehicle for their own genome, and in specific conditions, immunodeficiency among others, induce the expression of phage particles, which can be detected by the immune system [38]. Other bacteriophages modulate bacterial antigenicity through the production of enzymes capable of modifying the O-antigen component of lipopolysaccharide (LPS) in microorganisms such as salmonella,* E. coli*,* Shigella*, and* Vibrio cholera *[39–42].

In addition, as discussed by Cuesta et al. [43], bacteriophage proteins enhance the potency of DNA vaccines.

However, intestinal bacteriophages are able to circumvent the adaptive immunity of their hosts, thanks to hypervariable regions found within their genomes. These regions are known to collocate into genes that encode for phage tail-fibre proteins and immunoglobulin super-family (IgSF) proteins, which could act as scaffolds for the presentation of diversified phage peptide sequences. Although the physiological relevance of these hypervariable regions still remains to be clarified, it is plausible that such a diversity-generating mechanism could enable phages to evade the antibodies targeting the phage particles [44, 45].

### 3.3. Interaction between Eukaryotic Viruses and the Immune System

To date, scant information is available about the relationship between eukaryotic intestinal viruses and the host immune system. However, the few studies performed so far suggest that the eukaryotic virome could have a significant impact on host defence mechanisms against viral and/or bacterial pathogenic infections.

It has also been suggested that other viruses that chronically reside in the healthy tissue of individuals, such as Herpesviruses, Poliomaviruses, Adenoviruses, Papillomaviruses, Hepatitis B and C viruses, and Human Immunodeficiency Virus (HIV), can cause acute or latent infections that protect the host from further viral and bacterial infections [46]. Indeed, an interesting mutual symbiosis experiment has shown that chronic infection with a gamma-herpes virus increases resistance to both* Listeria monocytogenes* and* Yersinia pestis* in mice [46]. It is also known to activate natural killer (NK) cells, resulting in increased resistance to tumour grafts [47].

However, other chronic viral infections can bring about a reduction in host immunity and increase susceptibility to infection. In particular, pathogenic immunodeficiency viruses, including Simian Immunodeficiency Virus (SIV)—which causes AIDS in rhesus monkeys—have been associated with damage to the intestinal barrier, resulting in an expansion of the gut virome [28]. Chronic immune suppression inevitably results in global immune deficiency of the host, allowing select enteric viruses to damage the intestinal epithelial cells, in turn promoting translocation of enteric viruses, commensal bacteria, and bacterial antigens across the epithelial surface, resulting in inflammation and systemic infection [48].

## 4. The Gut Virome in Health and Disease

The realization that viruses in asymptomatic hosts do not always cause the death of infected cells has prompted the emergence of a paradigm wherein the virome independently influences the host, aside from the classical immune response triggered to fight disease [5]. Indeed, since studies on the human bacterial microbiome demonstrated the presence of mucosal viruses in healthy individuals, the traditional concept of viral infection has been overturned. It has been established that viruses are prevalent in the gastrointestinal tract, despite the absence of symptoms, which suggests that even in health the gut mucosa is characterized by frequent infections that become part the virome and may in turn bring beneficial and/or damaging effects on the host. It is likely, therefore, that the gut virome is able to influence the host phenotype during health, as well as inflammation and disease, by interacting with both other members of the gut microbiome and host genetics factors. In particular, phages may modulate host-bacterial interactions by infecting bacteria, and it is equally feasible that the gut bacteriome may regulate the gut virome [28].

### 4.1. From Dysbiosis to Chronic Disease through Inflammatory Pathways

The intestinal phages may contribute to the transition from health to disease by helping to bring about dysbiosis—an imbalance between symbiotic bacteria and pathobionts [49].

Although little data regarding the role of phage in shaping intestinal bacterial dysbiosis is available to date, de Paepe et al. have postulated several mechanisms by which commensal bacteriophages could affect the ecosystem of the gut microbiota [49].

One such mechanism, termed “*Kill the winner*” suggests that phages shape the intestinal bacterial microbiota through density-dependent predation. In other words, phages kill only the dominant commensal bacteria (the “winning” microorganisms) in the intestinal ecosystem, thereby reducing their numbers. Indeed, just such a relationship has been demonstrated by Reyes et al. in adult germ-free mice colonized with 15 symbiotic bacteria and infected with a cocktail of faecal phages [50]. Phage predation is also suggested by the presence of clustered regularly interspaced short palindromic repeat (CRISPR) systems in human commensal bacteria. CRISPR spacers recognize and silence exogenous genetic elements such as phages, thereby conferring a type of acquired immunity [28].

According to another potential mechanism, described as the “*biological weapon*” model, commensal bacteria would use their phages to kill another bacterial competitor for the intestinal environment [51, 52]. In this scenario, the phage would provide immunity to its carrier bacteria against further infection [53]. Acting as “*biological weapons*”, phages would cause massive lysis of competing microorganisms and a consequent shift in the composition of the population, leading to dysbiosis, and, in some cases, an inflammatory response [49]. Although this is an appealing hypothesis, further work is needed to confirm the existence of this mechanism.

Indeed, still other models have been put forward to explain the contribution of phages to intestinal dysbiosis. In one such model, so-called “*community shuffling*” [54], conditions of stress, such as antibiotic therapy, inflammation, and oxidative stress, have been theoretically implicated as triggers in prophage induction in several bacterial species like* E. coli *[55] and* Clostridium difficile* [56]. This theory is supported by the 30-fold increase in virus-like particles seen in biopsy specimens from patients with Crohn's disease with respect to healthy controls [57]. In the “*community shuffling*” model, this prophage induction would contribute to intestinal dysbiosis by altering the relationship between bacterial symbionts and pathobionts [54].

It is also feasible that temperate phages could affect the ecosystem without killing bacteria by carrying genes that modify bacterial phenotypes. This model, that is, the “*emergence of new bacterial strains*” has been demonstrated in* Escherichia coli* strain O104:H4, which can undergo lysogenic conversion, acquiring a Shiga-toxin encoding phage [58].

However it occurs, the intestinal dysbiosis promoted by the virome can be a triggering factor for inflammatory bowel disease (IBD) [59], Crohn's Disease (CD) [60], and colon cancer [61].

Inflammatory bowel diseases comprise a group of chronic inflammatory conditions that affect the gastrointestinal tract. This condition depends on individual genetic susceptibility, functional alterations in the intestinal epithelial barrier, dysbiosis, and immune factors.

As discussed by Lawlor and Moss [62] cytomegalovirus (CMV) is present in up to 70% of IBD patients, and that its reactivation could be associated with a type of colitis that displays some symptoms of IBD. Despite the implication of viral factors, it has been shown that antiviral treatment for CMV in IBD patients has no discernable impact on the outcome of the inflammatory disease [63]. It is therefore legitimate to ask whether CMV reactivation actively worsens the disease, or whether it is merely a “bystander” of inflammation [59].

Studies in mice deficient for the IBD susceptibility gene Atg16L1, which is involved in the autophagy pathway, have suggested a role for enteric viral infection in the pathogenesis of CD [16, 64–67]. Indeed, the Atg16L1 protein plays an important role in the biology of Paneth cells—specialized secretory cells located within the intestinal crypts—which release antimicrobial compounds and other substances that affect the gut microbiota [68]. Moreover, it found that intestinal noroviruses cause an abnormal phenotype of Paneth cell in mice with reduced expression of Atg16L1 (Atg16L1 hypomorphs), thereby highlighting an unexpected role of viruses in CD pathogenesis and showing how viral infection can have a profound influence on the expression of complex diseases [68].

## 5. Future Perspectives

In recent years, the development of high-throughput sequencing techniques has enabled partial characterization of the microbial composition of the healthy human gut, showing that viruses are important components of such communities (virome is described as follows). 

*Virome. Major Advances*

- The human virome is the collection of all viruses found in or on humans, including eukaryotic and prokaryotic viruses that cause acute, persistent, or latent infection, and viruses integrated into the human genome, such as endogenous retroviruses [1, 2]. It also contains viruses that infect plants, presumably taken in with the host diet [15, 17, 18].
- The virome has a profound impact on the composition and functional properties of the bacterial microbiota, which could in turn shape the development and function of the immune system [49].
- The gastrointestinal virome, by interacting with the mucus layer, epithelial cells, and underlying lamina propria immune cells, can contribute to the health or disease of the host [60].
- The enteric viruses are involved in the pathogenesis of dysbiosis and intestinal disorders, including inflammatory bowel disease (IBD), Crohn's disease (CD) [60], and colon cancer [61].
- The virome may contribute to phenotypic variation by regulating immunophenotype and the transcriptional state of the healthy host, reflecting their role in gene transfer and evolution [2].
- Phages may serve as important reservoirs of genetic diversity in the microbiota by acting as vehicles for the horizontal transfer of virulence, antibiotic resistance and metabolic determinants among bacteria [49].

This raises major questions for research, which is currently being focused on clarifying the qualitative and quantitative composition of the human intestinal virome.

Although it is generally taken for granted that the intestinal virome is mainly composed of eukaryotic viruses and bacteriophages, there are suggestions that the counts used to make this assertion may be erroneous. Indeed, metagenomic sequencing analyses have often ignored RNA viruses, and the isolation procedures currently in use may prevent detection of some viruses in gut virome samples [2].

Another question to be answered is how the virome populations shape the composition, functional properties, and antigenicity of commensal bacteria, and what repercussions such interactions may have on host immunity and health.

In particular, better identification of PAMPs and viromal antigens that stimulate innate and adaptative systems should provide further insight into how our immune system protects against pathogenic viral and/or bacterial infections.

We also need to ascertain whether virus-host interactions covertly influence other disease phenotypes. In this regard it is vital to search for hitherto undetected effects that common viral infections might have on the pathogenesis of complex human diseases, the effects of noroviruses on Crohn's disease being a case in point [3]. Indeed, a better understanding of the mechanisms by which host-virus interactions contribute to complex diseases may promote the development of new, more effective treatments.

The virome plays an important role in regulating the transcriptional state of a healthy host, which may respond differently to disease triggers, depending on the individual genetic constitution and virome composition of the latter. In this scenario, variations in the virome may contribute to phenotypic variation by regulating the immunophenotype, rather than by acting as pathogens.

Hence metagenetics—in essence the integrated study of the genetic impact on the host of the microbiome (and therefore virome) and vice versa* in vivo*—is set to become a major field of research.

Indeed, by helping us unravel the complex interactions between the virome and host genome, particularly as regards immunity, it is likely to shed considerable light on our genetics, health, and disease.
